# Supplementary material for: Splice-Junction-Based Mapping of Alternative Isoforms in the Human Proteome
Source: Cell Rep. Author manuscript; Available in PMC 2020 Jan 15. (PMC6961840; doi:10.1016/j.celrep.2019.11.026)
Supplement: 2 [file NIHMS1546469-supplement-2.zip › DF1/PXD010154/Heart/Heart_62_NEXN_AEQVNEDEENQDTAK.pdf]

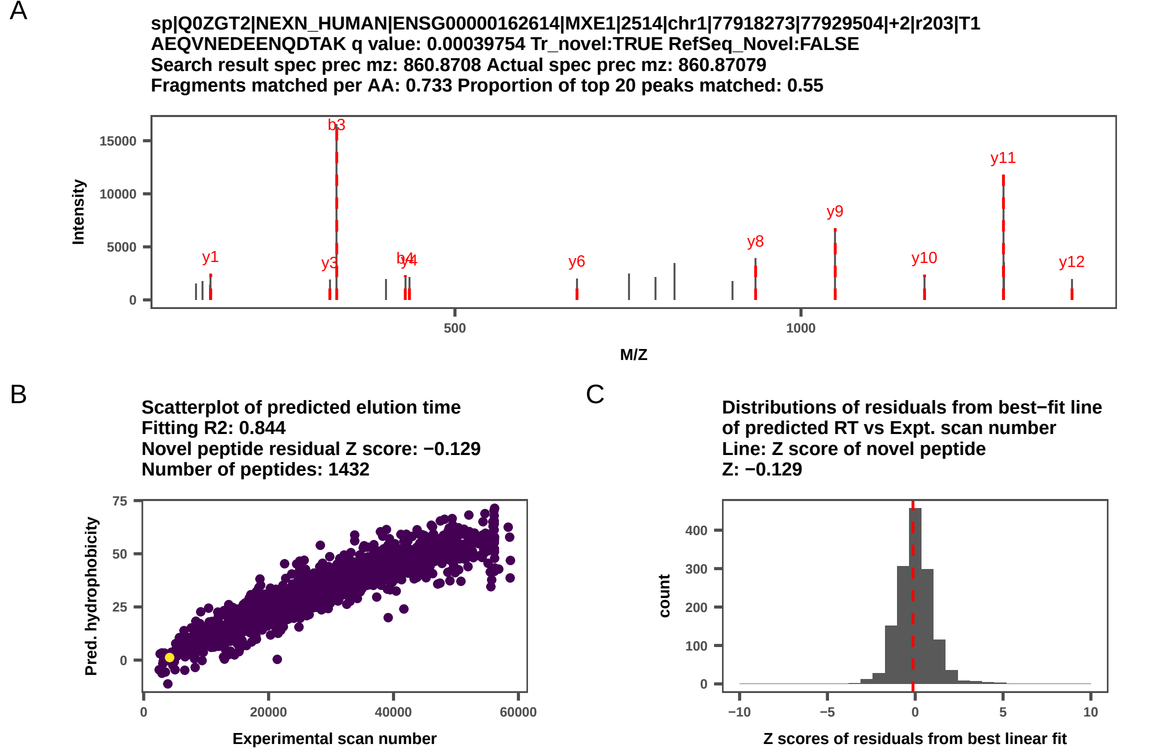
A
sp|Q0ZGT2|NEXN_HUMAN|ENSG00000162614|MXE1|2514|chr1|77918273|77929504|+2|r203|T1
AEQVNEDEENQDTAK q value: 0.00039754 Tr_novel:TRUE RefSeq_Novel:FALSE
Search result spec prec mz: 860.8708 Actual spec prec mz: 860.87079
Fragments matched per AA: 0.733 Proportion of top 20 peaks matched: 0.55
Intensity
15000
10000
5000
0
b3
y11
y9
y8
y1
y3
b4
y4
y6
y10
y12
500
1000
M/Z
B
Scatterplot of predicted elution time
Fitting R2: 0.844
Novel peptide residual Z score: −0.129
Number of peptides: 1432
Pred. hydrophobicity
75
50
25
0
0
20000
40000
60000
Experimental scan number
C
Distributions of residuals from best−fit line
of predicted RT vs Expt. scan number
Line: Z score of novel peptide
Z: −0.129
count
400
300
200
100
0
−10
−5
0
5
10
Z scores of residuals from best linear fit
